# The MtrAB two-component system controls antibiotic production in *Streptomyces coelicolor* A3(2)

**DOI:** 10.1099/mic.0.000524

**Published:** 2017-09-08

**Authors:** Nicolle F. Som, Daniel Heine, Neil Holmes, Felicity Knowles, Govind Chandra, Ryan F. Seipke, Paul A. Hoskisson, Barrie Wilkinson, Matthew I. Hutchings

**Affiliations:** ^1^​ School of Biological Sciences, University of East Anglia, Norwich Research Park, Norwich, NR4 7TJ, UK; ^2^​ Department of Molecular Microbiology, John Innes Centre, Norwich Research Park, Norwich, NR4 7UH, UK; ^3^​ School of Molecular and Cellular Biology, Astbury Centre for Structural Molecular Biology, University of Leeds, Leeds, LS2 9JT, UK; ^4^​ Strathclyde Institute of Pharmacy and Biomedical Sciences, University of Strathclyde, 161, Cathedral Street, Glasgow, G4 0RE, UK

**Keywords:** *Streptomyces*, antibiotics, sporulation, cryptic gene clusters

## Abstract

MtrAB is a highly conserved two-component system implicated in the regulation of cell division in the Actinobacteria. It coordinates DNA replication with cell division in the unicellular *Mycobacterium tuberculosis* and links antibiotic production to sporulation in the filamentous *Streptomyces venezuelae.* Chloramphenicol biosynthesis is directly regulated by MtrA in *S. venezuelae* and deletion of *mtrB* constitutively activates MtrA and results in constitutive over-production of chloramphenicol. Here we report that in *Streptomyces coelicolor*, MtrA binds to sites upstream of developmental genes and the genes encoding ActII-1, ActII-4 and RedZ, which are cluster-situated regulators of the antibiotics actinorhodin (Act) and undecylprodigiosin (Red). Consistent with this, deletion of *mtrB* switches on the production of Act, Red and streptorubin B, a product of the Red pathway. Thus, we propose that MtrA is a key regulator that links antibiotic production to development and can be used to upregulate antibiotic production in distantly related streptomycetes.

The multicellular filamentous bacteria in the genus *Streptomyces* have complex life cycles and make numerous specialized metabolites, including more than half of all known antibiotics [1]. Most of these antibiotics were discovered more than 50 years ago, but the alarming rise in antimicrobial resistance over the last five decades has driven a resurgence of interest in *Streptomyces* natural products in the 21st century. This has largely been driven by genome sequencing, which has revealed that up to 90 % of the specialized metabolites encoded by *Streptomyces* strains are not produced under laboratory conditions [2]. The environmental signals and signal transduction systems controlling expression of their specialized metabolite biosynthetic gene clusters (BGCs) are poorly understood, which is why most of them remain cryptic. However, antibiotic production has long been known to be linked to the differentiation of actively growing substrate mycelium into aerial mycelium and spores, the equivalent of cell division in unicellular bacteria [1]. Identification and manipulation of the global signalling pathways that control these processes could therefore enable the discovery of new and useful natural products, or be used to make antibiotic overproducing strains for industry. To this end, we recently characterized a two-component system called MtrAB in *Streptomyces venezuelae* [3]. The MtrAB two-component system is highly conserved in the phylum *Actinobacteria* and is best characterized in *M. tuberculosis*, where it coordinates DNA replication with cell division [4, 5]. We reported that MtrA coordinates antibiotic production with sporulation and that deletion of the sensor kinase gene *mtrB* results in constitutively active MtrA and constitutive high-level production of chloramphenicol, as well as a global shift in the metabolome of *S. venezuelae* [3].

In this work, we characterized MtrAB in another streptomycete, *Streptomyces coelicolor*, a model species that makes the pigmented antibiotics actinorhodin (Act) and undecylprodigiosin (Red). The 16S rDNA phylogenetic tree of the family *Streptomycetaceae* shows that *S. venezuelae* (clade 40) is highly divergent from *S. coelicolor* (clade 112), which is why we chose to characterize the system in these distantly related species [6]. Unlike *S. venezuelae*, *S. coelicolor* does not sporulate in liquid culture, but grows as a vegetative mycelium, and this further enabled us to examine the role of MtrA during vegetative growth. We previously isolated an in-frame unmarked ∆*mtrA* mutant in *S. coelicolor* and reported that expression of the *mce* operon is reduced in this background [7]. For this study, we made further in-frame deletions in the *mtrB* and *lpqB* genes using Redirect PCR targeting and Flp recombinase [8] (Table S1, available in the online Supplementary Material). LpqB is an accessory lipoprotein that interacts with and reduces MtrB activity in *M. smegmatis*, and deletion of *lpqB* results in a filamentous strain that is reminiscent of streptomycetes and suggestive of a defect in cell division [9]. In-frame deletion of *S. coelicolor lpqB* had no visible effect on growth or development, but in-frame deletion of *mtrB* resulted in a small colony phenotype and a delay in sporulation, as judged by visible late production of the brown WhiE spore pigment in these colonies (Fig. S1). *In trans* complementation was attempted by introducing the relevant gene into the phiBT1 site on the integrative vector pMS82, under the control of the *mtrA* operon promoter [10]. This restored the wild-type phenotype to the *∆mtrB* mutant (Fig. S1).

Unlike *S. venezuelae*, *S. coelicolor* does not sporulate in liquid culture, but grows as a vegetative mycelium, and this allowed us to use chromatin immunoprecipitation followed by sequencing (ChIP-seq) to identify MtrA targets in vegetatively growing *Streptomyces* and to compare these targets to those identified in differentiating *S. venezuelae*. To determine where MtrA binds on the *S. coelicolor* genome we introduced a construct expressing MtrA-3xFlag under the control of the *mtrA* promoter into the phiBT1 site of the previously isolated *∆mtrA* mutant and performed ChIP-seq on cultures of this strain grown for 16 and 20 h in liquid maltose–yeast extract/malt extract (MYM) medium [11] with the wild-type as a control. ChIP-seq was performed as described previously and Bowtie was used to generate plots that could be visualized using Integrated Genome Browser [12, 13]. A full list of targets for the 16 and 20 h samples are given in Table S2 (NCBI Geo database accession number: GSE84311). The developmental and secondary metabolism genes bound by MtrA in both *S. venezuelae* NRRL B-65442 and *S. coelicolor* M145 are listed in Table 1. Many of the developmental genes bound by MtrA in *S. venezuelae* were not enriched in the *S. coelicolor* data, most likely because *S. coelicolor* is growing vegetatively and most specialized metabolite BGCs are not conserved between these species [3]. It is interesting that the promoter of the *ectABCD* operon is highly enriched in MtrA ChIP-seq experiments in both *S. coelicolor* and *S. venezuelae*. In the latter it was the most highly enriched target in the entire dataset [3]. This BGC encodes for the osmolytes ectoine and 5′ hydroxyectoine, but we could not detect either compound in the wild-type or *∆mtrB* strains, suggesting that MtrA may repress *ectABCD.* One of the conserved targets that is particularly worth noting is CdgB, which makes the secondary messenger cyclic di-GMP that controls the activity of the master regulator of differentiation BldD [14]. The *bldD* gene is an MtrA target in *S. coelicolor* but not in *S. venezuelae*, at least under the conditions used for these experiments (Table S2)[3]. Additional conserved targets include WhiB, WhiD and WblE, which are all members of the WhiB-like (Wbl) family of Fe–S-containing transcription factors that are restricted to actinobacteria (Fig. 1a and Table 1). WhiB and WhiD regulate early- and late-stage sporulation, respectively [15, 16]. WblE is essential in *S. coelicolor* and *M. tuberculosis*, and although its function is still unknown this suggests that it must play a key role in their life cycles [17].

**Table 1.** Developmental and secondary metabolism genes bound by MtrA in vegetatively growing *Streptomyces coelicolor* M145 and differentiating *Streptomyces venezuelae* NRRL B-65442 [3]

**Gene name**	**Gene number**	**Function**	**Reference**
*cdgB*	*sco4281*	Cyclic di-GMP metabolism	[21]
*bldM*	*sco4768*	Orphan RR, forms homo- and heterodimers with WhiI to regulate differentiation, encoded divergently from *whiD.*	[22, 23]
*chpF*	*sco2705*	Surfactant required for aerial hyphae formation	[24]
*sapB*	*sco6682*	Surfactant required for aerial hyphae formation	[25]
*filP*	*sco5396*	Filament forming protein involves in hyphal growth	[26]
*ftsZ*	*sco2082*	FtsZ is a tubulin homologue and forms Z rings to mark the sites of cell division	[27]
*smc*	*sco5577*	Structural maintenance of chromosomes	[28]
*wblE*	*sco5240*	Essential WhiB-like (Wbl) protein and transcription factor	[17]
*whiB*	*sco3034*	Wbl protein that regulates early-stage sporulation	[29]
*whiD*	*sco4767*	Wbl protein that regulates late sporulation, encoded divergently from *bldM*	[15]
*whiI*	*sco6029*	Orphan RR, forms heterodimers with BldM to regulate differentiation	[23]
*ectABCD*	*sco1864*	Biosynthesis of the secondary metabolites ectoine and 5′ hydroxyectoine	[30]

**Fig. 1.**
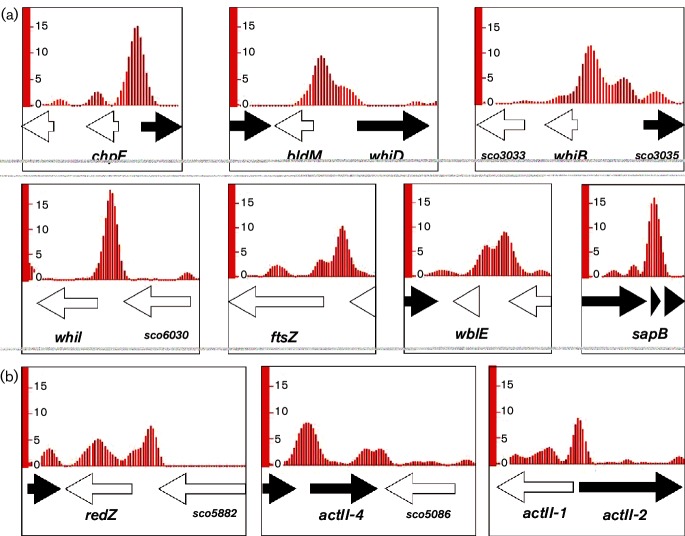
(a) MtrA ChIP peaks upstream of the *S. coelicolor* M145 developmental genes *chpF*, the divergent *bldM* and *whiD*, *whiB*, *whiI*, *ftsZ*, *wblE* and *sapB*. (b). MtrA ChIP peaks upstream of the cluster-situated regulatory genes *redZ* (undecylprodigiosin), *actII-1* and *actII-4* (actinorhodin). The *y-*axis gives the enrichment value relative to the surrounding region of 4000 nucleotides.

*S. coelicolor* is the best characterized *Streptomyces* species in terms of its specialized metabolites and their BGCs [18, 19], and the ChIP-seq data show that genes encoding the cluster-specific regulators for Act and Red are enriched (Fig. 1b). RedZ and ActII-4 are activators and ActII-1 is a repressor. The divergently encoded ActII-2 is a putative transporter for Act. The *∆mtrB* mutant produces more pigments than the wild-type, suggesting that MtrA may activate production of Act and/or Red (Figs 2 and S1). To test this, we grew the *S. coelicolor* wild-type and ∆*mtrB* mutants in biological triplicates and extracted the whole broth with methanol. The resulting supernatants were analysed by UPLC-HRMS (see the Supplementary Material for the methods used) and the results showed that while the *S. coelicolor ∆mtrB* mutant produces Act and Red, they are not detectable in the wild-type strain in liquid medium (Fig. 2). Given that MtrA is likely to be constitutively active in the absence of MtrB, it is possible that MtrA directly activates the production of Act and Red. We did not perform expression studies on the *∆mtrA* mutant because we could not fully complement the mutation. However, we have confidence in the ChIP-seq data because MtrA-3xFlag rescues an *S. venezuelae ∆mtrA* mutant and because many of the MtrA targets we identified in *S. coelicolor ∆mtrA*+MtrA-3xFlag are conserved MtrA targets in *S. venezuelae* (Table 1) [3]. We also detected significant amounts of streptorubin B in the *∆mtrB* cultures, a specialized metabolite encoded by the Red biosynthetic pathway [20]. The production of the siderophores desferrioxamine B and E is reduced in the *∆mtrB* mutant and we could not detect germicidin A (Fig. 2), although the BGCs encoding the production of these compounds are not bound by MtrA (Table S2).

**Fig. 2.**
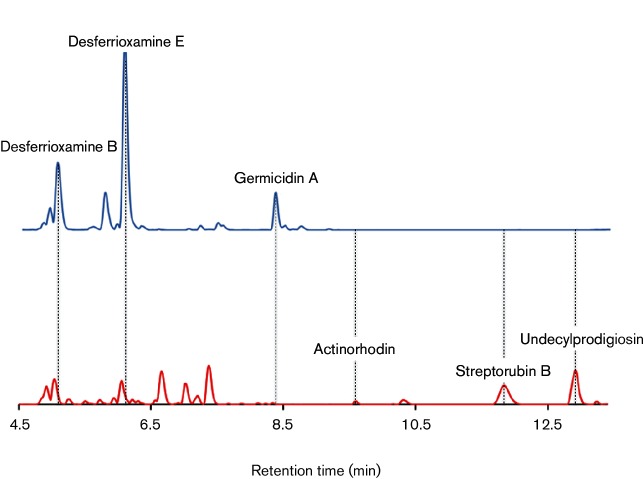
Representative UPLC-HRMS traces of culture extracts of wild-type *S. coelicolor* M145 (top) and the isogenic *ΔmtrB* mutant (bottom) are shown for comparison. The *y*-axes represent the total ion count and are normalized. The *x*-axis indicate retention time and refers to both traces. The siderophores desferrioxamines A and B were down-regulated and germicidin A was not detected in the *ΔmtrB* mutant, while actinorhodin, undecylprodigiosin and streptorubin B were produced in the absence of MtrB but were not detectable in the wild-type extracts.

In conclusion, we have demonstrated that the MtrAB two-component system helps control antibiotic production in the distantly related *S. coelicolor* and *S. venezuelae*, and binds to developmental genes in both vegetatively and developmentally growing species. We have also shown that deletion of *mtrB* or manipulation of MtrA activity can be used to increase antibiotic production in these *Streptomyces* species. Given the fact that MtrAB is conserved in all *Streptomyces* species and in other filamentous actinomycetes, we suggest that manipulation of MtrA activity could be a general tool for upregulating antibiotic production in these bacteria.

## Supplementary Data

421 Supplementary File 1

## Supplementary Data

357 Supplementary File 2
