# Supplementary material for: The MtrAB two-component system controls antibiotic production in Streptomyces coelicolor A3(2)
Source: Microbiology (Reading). 2017 Sep 8;163(10):1415–9. doi: 10.1099/mic.0.000524 (PMC5845573; doi:10.1099/mic.0.000524)
Supplement: Supplementary File 1 [file mic-163-1415-s001.pdf]

SUPPLEMENTARY INFORMATION

The MtrAB two component system controls antibiotic production in *Streptomyces coelicolor* A3(2)

Nicolle F. Som[1#], Daniel Heine[2#], Neil Holmes[1], Felicity Knowles[1], Govind Chandra[2], Ryan F. Seipke[3], Paul A. Hoskisson[4], Barrie Wilkinson[2*] and Matthew I Hutchings[1*]

# Authors contributed equally;
* Correspondence: m.hutchings@uea.ac.uk and barrie.wilkinson@jic.ac.uk

[1]School of Biological Sciences, University of East Anglia, Norwich Research Park, Norwich, United Kingdom. NR4 7TJ.

[2]Department of Molecular Microbiology, John Innes Centre, Norwich Research Park, Norwich, United Kingdom. NR4 7TJ.

[3] School of Molecular & Cellular Biology, Astbury Centre for Structural Molecular Biology, University of Leeds, Leeds, LS2 9JT, UK

[4]Strathclyde Institute of Pharmacy and Biomedical Sciences, University of Strathclyde, 161, Cathedral Street, Glasgow, G4 0RE, UK

TABLE OF CONTENTS

Figure S1. Colony morphologies of the wild-type, ∆*mtrA* ∆*mtrB* and ∆*lpqB S. coelicolor* M145 strains.

Table S1. Strains, plasmids and primers used in this study.

Analytical Chemistry Methods

ChIP-seq Methods and Table headings

Table S2. Excel spreadsheet listing MtrA binding sites mapped on the *S. coelicolor* M145 using ChIP-seq.

Figure S1. Representative colonies of wild-type (WT) *Streptomyces coelicolor* M145 and the isogenic, in-frame Δ*mtrB* and Δ*lpqB* mutants without or with +*mtrB* and +*lpqB*) *in trans* copies of the relevant gene integrated in single copy into the phiBT1 phage integrations site using pMS82 and under the control of the *mtrAB-lpqB* operon promoter. Strains were grown for 3 or 5 days on MS agar (20g mannitol, 20g soya flour, 20g agar in 1L tap water, autoclaved twice) as indicated.

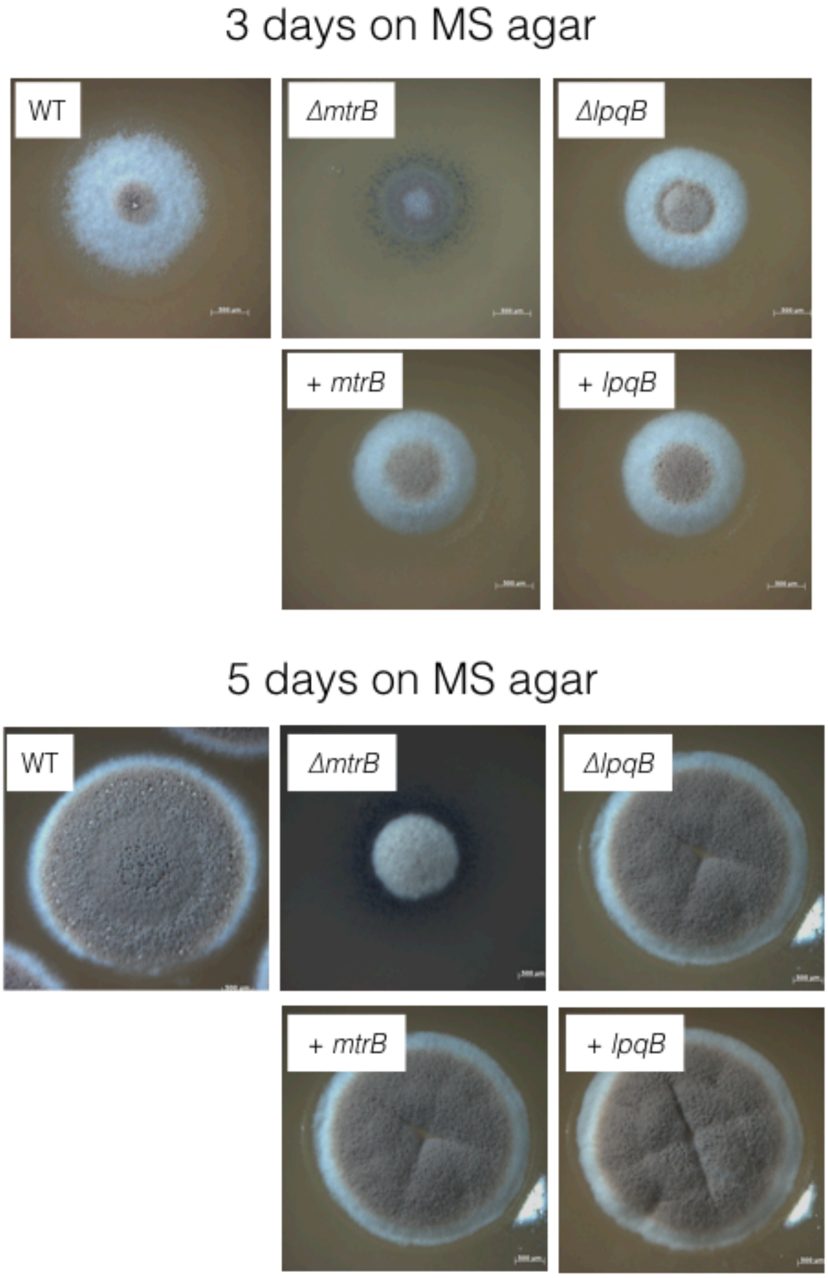

Table S1. Strains and plasmids used in this study – available on request to m.hutchings@uea.ac.uk.

**Bacterial strain**	**Genotype**	**Source**
S. coelicolor M145	SCP1^{-} SCP2^{-} Pgl+	John Innes Centre, Norwich, UK.
ΔlpqB	M145Δ*lpqB* (in frame deletion)	This Study
ΔmtrB	M145Δ*mtrB* (in frame deletion)	This Study
ΔmtrA	M145Δ*mtrA* (in frame deletion)	(1)
ΔlpqB phiBT1-lpqB	M145Δ*lpqB* + pMS82/*lpqB*	This Study
ΔmtrB phiBT1-mtrB	M145Δ*mtrB* + pMS82/*mtrB*	This Study
ΔmtrA phiBT1-mtrA-3xFlag	M145Δ*mtrA* + pNS109	This Study
**Cosmid or Plasmid**	**Description**	**Source**
E33	Supercos1 with an insert of the *S. coelicolor* M145 genome containing genes *sco3000* to *sco3022* (*mtrAB-lpqB* are gene numbers *sco3013-11*)	John Innes Centre, Norwich, UK. (2)
E33Δ*lpqB*	E33 with an in-frame deletion in *lpqB*	This Study
E33Δ*mtrB*	E33 with an in-frame deletion in *mtrB*	This Study
E33Δ*mtrA*	E33 with an in-frame deletion in *mtrA*	(1)
pMS82	Phage vector that integrates into the phiBT1 site	(3)
pMS82/*lpqB*	pMS82 with the *lpqB* gene cloned downstream of the *sco3014-mtrAB-lpqB* operon promoter	This Study
pMS82/*mtrB*	pMS82 with the *mtrB* gene cloned downstream of the *sco3014-mtrAB-lpqB* operon promoter	This Study
pNS109	pMS82 *mtrAp mtrA*-3xFlag	(4)

Analytical chemistry. *S. coelicolor* M145 and isogenic Δ*mtrB* strains were grown in biological triplicates and then 750 µL of each culture was mixed with 250 µL methanol. After 10 minutes the samples were centrifuged at 12.000×g/min and the supernatant was used for UPLC-HRMS analysis. Measurements were performed on a Nexera X2 liquid chromatograph (LC-

30AD) LCMS system (Shimadzu) connected to an autosampler (SIL-30AC), a Prominence column oven (CTO-20AC) and a Prominence photo diode array detector (SPD-M20A). The UPLC-System was connected to an LCMS-IT-TOF Liquid Chromatograph mass spectrometer (Shimadzu). We used a Kinetex® 1.7 µm C18 100 Å, 100×2.1 mm column (Phenomenex) and applied a gradient of water (0.1% formic acid)/methanol. Starting conditions: 90/10, hold at 90/10 for 1 min, to 0/100 within 9.00 min, hold for 2.00 min, to 90/10 from within 0.5 min, hold at 90/10 for 0.5 min. All solvents for analytical UPLC-HRMS were obtained commercially at least in HPLC grade from Fisher Scientific and were filtered prior to use. Formic acid (0.1%) was added to the water.

ChIP-seq Methods. The reads in the fastq files received from the sequencing contractor were aligned to the *Streptomyces coelicolor* M145 genome using the bowtie2 software, which resulted in one SAM (.sam) file for each fastq file. All further operations described below were carried out using a combination of Perl scripts dependent on the BioPerl toolkit and R scripts. From each SAM file, coverage at (number of reads mapping to) each nucleotide position of the *Streptomyces coelicolor* genome was calculated and the output was saved in files referred to as coverage files. For each coverage file, a local enrichment was calculated in a moving window of 51 nucleotides (nt) moving in steps of 25 nucleotides as (the sum of coverage at each nucleotide position in the 51-nt window) divided by (the sum of coverage at each nucleotide position in a 4,001-nucleotide window centred around the 51-nucleotide window). This results in an enrichment ratio value for every 25 nucleotides along the genome.

All nucleotide positions where the enrichment ratio was less than 1.5 were removed and then a negative binomial distribution was fitted to the data using the fitdistr function of the MASS package in R. Thus, we could arrive at the size and the mu parameters of the binomial distribution.

The values of size and mu parameters resulting from the fitting of the binomial distribution were then used to calculate the P-values for each enrichment ratio using the pnbinom function of R. Finally the P-values were adjusted for multiple testing by using the p.adjust function of R using the Benjamini and Hochberg method. This resulted in tables which had three columns: Genomic position, Enrichment ratio and, Adjusted P-value.

Using the coordinates of the genes in *Streptomyces coelicolor*, information about genes on the left and right of each genomic position was added to the tables made above.

The columns in Table S2 are:

1. Position on the genome.
2. Enrichment relative to the surrounding region of 4000 nucleotides.
3. P-value.
4. Gene to the left of the position in column 1.
5. The direction of this gene.
6. Distance from the start of this gene to the position in column 1.
7. Annotated product of this gene.

Columns 8, 9, 10, and 11 are similar to 4, 5, 6 and 7 but they are for the gene inside which the position in column 1. lies. These can be all "-" (dashes) if the position is not within a gene.

Columns 12, 13, 14, and 15 are similar to 4, 5, 6 and 7 but they are for the gene on the right of the position in column 1.

References

1. Clark LC, Seipke RF, Prieto P, Willemse J, van Wezel GP, Hutchings MI, et al. Mammalian cell entry genes in Streptomyces may provide clues to the evolution of bacterial virulence. Sci Rep. 2013 3:1109.

2. Redenbach, Kieser HM, Denapaite D, Eichner A, Cullum J, Kinashi H, et al. A set of ordered cosmids and a detailed genetic and physical map for the 8 Mb Streptomyces coelicolor A3(2) chromosome. 1996 21:77–96.

3. Gregory MA, Till R, Smith MCM. Integration site for Streptomyces phage phiBT1 and development of site-specific integrating vectors. J Bacteriol. 2003 185:5320–3.

4. Som NF, Heine D, Munnoch JT, Holmes NA, Knowles F, Chandra G, et al. MtrA is an essential regulator that coordinates antibiotic production and sporulation in Streptomyces species. bioRxiv. 2016 doi:https://doi.org/10.1101/090399.
